# Wünderlich Syndrome Without Classic Signs: A Case of Spontaneous Retroperitoneal Hemorrhage From a Ruptured Renal Angiomyolipoma

**DOI:** 10.7759/cureus.106094

**Published:** 2026-03-29

**Authors:** César Andino-Colón, Axel Nuñez, Yolymar Poventud, Joanna Mercado

**Affiliations:** 1 Emergency Medicine, University of Puerto Rico, Medical Sciences Campus, San Juan, PRI

**Keywords:** emergency medicine, renal angiomyolipoma, spontaneous retroperitoneal hemorrhage, transcatheter arterial embolization, wunderlich syndrome

## Abstract

Wünderlich syndrome is a non-traumatic retroperitoneal hemorrhage confined to the subcapsular, perirenal, and/or pararenal space. It has been previously associated with Lenk's triad of flank pain, hematuria, and hypovolemic shock; however, this triad is not always present, making early recognition in the emergency department challenging. We describe a 39-year-old male who presented with sudden-onset severe left flank pain, diaphoresis, and persistent tachycardia without hematuria or hypotension. Contrast-enhanced computed tomography of the abdomen and pelvis rapidly showed a left renal angiomyolipoma with fat and extensive active retroperitoneal hemorrhage. Definitive care required interfacility transfer for angiography and transcatheter arterial embolization, during which active extravasation was identified from adrenal arterial branches. Despite aggressive resuscitative and endovascular measures, the patient experienced progressive hemodynamic deterioration and expired. This case reinforces that early cross-sectional imaging is essential for diagnosing spontaneous retroperitoneal hemorrhage, enabling timely escalation to interventional management. Prompt recognition, rapid imaging, and expedited access to definitive intervention remain critical determinants of outcome.

## Introduction

Wünderlich syndrome is a spontaneous hemorrhage confined to the subcapsular or perirenal space [1,2]. This rare, life-threatening condition can result in significant retroperitoneal blood loss and hemodynamic instability if not promptly recognized [1,2]. It is classically associated with Lenk's triad of acute flank pain, palpable mass, and hypovolemic shock; however, the complete triad is present in only approximately 20% of cases [3-5]. Most patients instead present with isolated flank pain or nonspecific symptoms, and hematuria may be absent despite substantial hemorrhage [3,4].

Imaging plays a central role in establishing the diagnosis. Ultrasonography is a rapid bedside screening tool commonly used in the emergency department to assess for intraperitoneal free fluid in patients with undifferentiated abdominal or flank pain [6,7]. However, its ability to detect retroperitoneal hemorrhage is limited, and it may fail to identify perirenal bleeding or other retroperitoneal pathology [8,9].

Contrast-enhanced CT is the preferred imaging modality for the evaluation of suspected retroperitoneal hemorrhage, demonstrating high sensitivity for detecting hematomas and high accuracy in defining their extent and identifying active contrast extravasation with sensitivity approaching 95-100% [10,11]. It also enables identification of the underlying etiology of hemorrhage [10,11].

Renal neoplasms represent the most common etiology of Wünderlich syndrome, with angiomyolipoma being the most frequent individual entity, accounting for approximately 11-24% of spontaneous perirenal hemorrhages [3,4]. Angiomyolipomas are benign mesenchymal tumors composed of dysmorphic blood vessels, smooth muscle, and adipose tissue [12]. Despite their benign classification, they are highly vascular, and the risk of spontaneous rupture increases with tumor size and aneurysmal features [12-14].

Other etiologies include renal cystic disease, vascular abnormalities, infection, anticoagulation, and chronic kidney disease [4,5]. Clinical presentation is often variable and may not correlate with severity, as significant retroperitoneal bleeding may occur without hematuria or overt instability [3,4]. We present a case of Wünderlich syndrome secondary to a ruptured renal angiomyolipoma, emphasizing that early contrast-enhanced CT is critical, as initial clinical stability may obscure life-threatening retroperitoneal hemorrhage.

## Case presentation

A 39-year-old male with a history of hypertension presented to the emergency department at 02:00 with sudden-onset left flank pain that began at approximately 00:00, which awakened him from sleep. The pain was constant, sharp, non-radiating, and worsened with minimal movement. He denied trauma, prior similar episodes, hematuria, dysuria, hematochezia, fever, chest pain, nausea, or vomiting. There was no history of renal disease, malignancy, anticoagulant use, or connective tissue disorders.

On arrival, the patient appeared diaphoretic and in significant distress. Vital signs demonstrated blood pressure of 173/146 mmHg, heart rate of 133 bpm, respiratory rate of 18 breaths per minute, oxygen saturation of 96% on room air, and temperature of 36.7 °C. Abdominal examination revealed distention with diffuse tenderness and voluntary guarding, more pronounced over the left abdomen, without periumbilical or flank ecchymosis. Point-of-care ultrasonography did not demonstrate intra-abdominal free fluid.

**Figure 1:**
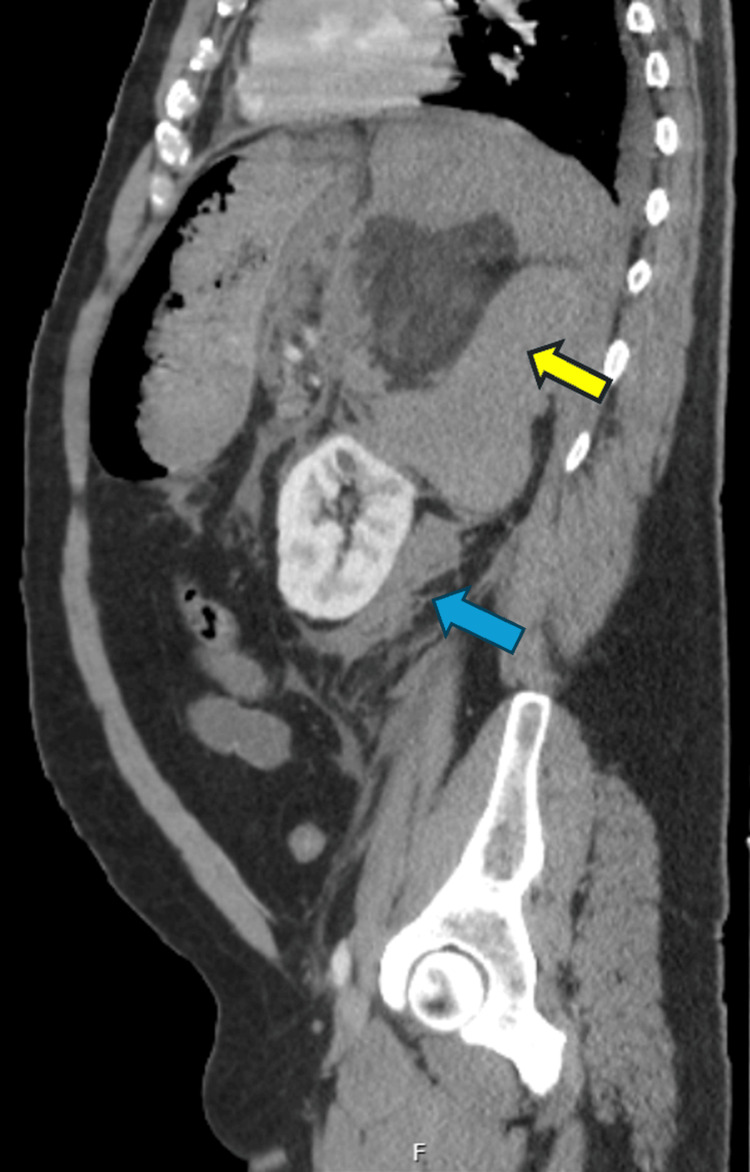
Renal angiomyolipoma Sagittal view of contrast-enhanced computed tomography of the abdomen and pelvis in the soft tissue window showing a large mass in the left kidney/suprarenal region containing soft tissue component, fat, and hemorrhage (yellow arrow). There is also evidence of moderate hemorrhage extending to the retroperitoneum, causing mass effect on the left kidney (blue arrow), spleen, and pancreas, concerning for a large angiomyolipoma with active hemorrhage to the right anterior pararenal space and pelvis.

**Figure 2:**
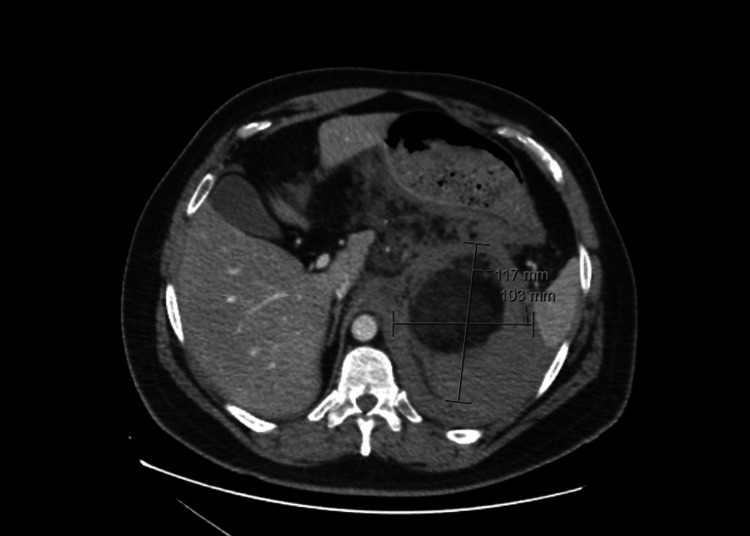
Computed tomography demonstrating renal angiomyolipoma Axial view of contrast-enhanced computed tomography of the abdomen and pelvis demonstrating a large heterogeneous left renal mass consistent with angiomyolipoma, measuring approximately 11.7 × 10.3 × 16.7 cm (calipers)

Initial laboratory evaluation at 02:37 demonstrated leukocytosis with a white blood cell count of 23.10 × 10³/µL, hemoglobin of 14.4 g/dL, hematocrit of 42.8%, and platelet count of 330 × 10³/µL. Basic metabolic panel revealed sodium of 135 mmol/L, potassium of 3.7 mmol/L, glucose of 224 mg/dL, creatinine of 1.09 mg/dL, and a blood urea nitrogen of 19. Coagulation studies demonstrated a prothrombin time of 12.9 seconds and a partial thromboplastin time of 26.6 seconds. Lipase was 50 U/L, and alkaline phosphatase was 94 U/L. Computed tomography angiography was not available at the presenting facility, and contrast-enhanced computed tomography of the abdomen and pelvis was obtained. Imaging reported at 05:30 demonstrated a large heterogeneous mass arising from the left kidney or suprarenal region measuring 11.7 × 10.3 × 16.7 cm, containing soft tissue, fat, and hemorrhagic components, with extensive retroperitoneal hemorrhage extending into the perirenal and pararenal spaces and exerting mass effect on adjacent structures (Figure 1). Findings were consistent with a ruptured angiomyolipoma (Figure 2). Additional findings included bilateral renal cysts without hydronephrosis or nephrolithiasis.

Urology and general surgery were consulted; however, given concern for retroperitoneal hemorrhage with an arterial source requiring endovascular management, definitive intervention was not available at the presenting facility. Transfer to a tertiary care center was arranged.

During his initial ED course prior to transfer, the patient received three liters of intravenous 0.9% normal saline, morphine for analgesia, ondansetron for nausea, and piperacillin-tazobactam for broad-spectrum antibiotics. Despite these interventions, he remained tachycardic. Repeat vital signs at 05:33 demonstrated blood pressure of 113/66 mmHg and heart rate of 118 bpm, with oxygen saturation of 100% on room air.

While en route to the receiving facility, repeat laboratory evaluation reported at the presenting facility at 08:55 showed worsening leukocytosis with a white blood cell count of 30.5 × 10³/µL and a decline in hemoglobin to 10.7 g/dL, with a hematocrit of 32.7%, representing a rapid decrease from the initial hemoglobin of 14.4 g/dL. However, the patient was en route to the tertiary facility at that moment. 

Vital signs recorded at 09:40 after the patient arrived at the tertiary facility demonstrated blood pressure of 92/63 mmHg, heart rate of 125 bpm, respiratory rate of 16 breaths per minute, temperature of 36.7 °C, and oxygen saturation of 99% on room air. He was admitted with an interventional radiology consultation for definitive management. The patient was placed on NPO status and continued on intravenous hydration, analgesia, and broad-spectrum antibiotics. Electrocardiogram and chest radiography were ordered as part of the initial evaluation.

Laboratory studies at 10:50 demonstrated hemoglobin of 9.7 g/dL, hematocrit of 30.1%, white blood cell count of 26.92 × 10³/µL, platelet count of 327 × 10³/µL, creatinine of 1.85 mg/dL, carbon dioxide of 21 mmol/L, and blood urea nitrogen of 23 mg/dL. Electrolytes revealed sodium of 140 mmol/L, potassium of 4.2 mmol/L, chloride of 108 mmol/L, and calcium of 8.3 md/dL.

At 14:08, vital signs demonstrated transient improvement with blood pressure of 99/63 mmHg and heart rate of 99 bpm. However, by 15:10, the patient developed recurrent tachycardia with a heart rate of 130 bpm and a respiratory rate of 27 breaths per minute.

The patient underwent emergent angiography beginning at 15:41. Intra-procedural vital signs at 15:44 demonstrated hypotension with blood pressure of 88/55 mmHg and tachycardia with heart rate of 136 bpm, requiring initiation of vasopressor support with norepinephrine. An abdominal aortogram demonstrated normal aortic anatomy without evidence of active extravasation. Selective catheterization of the left adrenal artery revealed a hypervascular mass with active contrast extravasation. A microcatheter was advanced into a feeding arterial branch, and superselective embolization was performed using 355 µm polyvinyl alcohol particles until stasis was achieved. Completion digital subtraction angiography confirmed cessation of extravasation with no residual tumor blush. Additional angiographic evaluation of the left inferior phrenic and left renal arteries demonstrated no contribution to the hemorrhage. Hemostasis was achieved using an Angio-Seal closure device, and the patient was transferred in stable condition to the holding area.

Post-procedural laboratory evaluation at 16:45 demonstrated hemoglobin of 9.9 g/dL and platelet count of 208 × 10³/µL. However, by 18:50, hemoglobin acutely declined to 5.7 g/dL, with a hematocrit of 18.4% and platelet count of 149 × 10³/µL, indicating ongoing hemorrhage despite embolization. Laboratory evaluation also revealed sodium of 147 mmol/L, potassium of 2.9 mmol/L, calcium of 3.8 mg/dL, chloride of 131 mmol/L, carbon dioxide less than 11 mmol/L, and creatinine of 1.08 mg/dL.

Arterial blood gas at 20:25 demonstrated severe acidemia with a pH of 7.12, pCO₂ of 61 mmHg, bicarbonate of 19 mmol/L, and base deficit of −11.0 mmol/L. Metabolic derangements were addressed, including administration of intravenous sodium bicarbonate.

At 20:40, the patient remained clinically unstable with a temperature of 35.6 °C, heart rate of 140 bpm, respiratory rate of 17 breaths per minute, blood pressure of 150/103 mmHg, and oxygen saturation of 100% while receiving supplemental oxygen. The patient received four units of packed red blood cells, with hemoglobin improving to 11.0 g/dL at 21:45. Despite this, he remained tachycardic and continued to demonstrate progressive hemodynamic instability.

Repeat angiographic evaluation was performed at 23:12 via left common femoral access. Selective catheterization with digital subtraction angiography of the left adrenal arteries, left inferior phrenic artery, and celiac axis with splenic artery did not demonstrate active extravasation. Given the absence of a surgically identifiable source of bleeding and the retroperitoneal nature of the hemorrhage, the condition was not amenable to operative management.

The patient was subsequently managed in the intensive care setting with continued hemodynamic support, including vasopressor therapy. Despite aggressive resuscitation, he developed refractory shock. During his ICU course, the patient developed worsening metabolic acidosis, respiratory failure, and hemodynamic instability despite escalating support. Approximately 27 hours after initial presentation, the patient developed cardiopulmonary arrest. Advanced cardiac life support was initiated but was unsuccessful, and the patient was pronounced deceased.

Table 1 shows the clinical timeline.

**Table 1:** Clinical timeline, hemodynamic progression, and key interventions

Time	Phase	Key Findings
00:00	Symptom onset	Sudden-onset left flank pain.
02:00	Initial ED evaluation	BP 173/146 mmHg, HR 133 bpm; Hgb 14.4 g/dL
02:00–05:30	Initial ED management	Intravenous fluids, analgesia, antiemetics, antibiotics. Persistent tachycardia.
05:30	Diagnostic imaging	CT demonstrated a large hemorrhagic renal mass with extensive retroperitoneal bleeding, consistent with a ruptured angiomyolipoma.
05:33	Reassessment	BP 113/66 mmHg, HR 118 bpm
07:52	Case accepted at tertiary facility	
8:30	Patient en route to tertiary facility	
08:55	Laboratory report at presenting facility	Hgb decreased to 10.7 g/dL, consistent with ongoing hemorrhage
09:40	Arrival at the receiving facility	BP 92/63 mmHg, HR 125 bpm
10:50	Laboratory progression	Hgb 9.7 g/dL, Cr 1.85 mg/dL
14:08	Serial vital signs	BP 99/63 mmHg, HR 99 bpm
15:10	Clinical deterioration	Recurrent tachycardia (HR 130 bpm)
15:41–16:20	First embolization	Active arterial bleeding identified and embolized
15:44	Intra-procedural instability	BP 88/55 mmHg, HR 136 bpm; vasopressors initiated
16:45	Post-procedure laboratories	Hgb 9.9 g/dL
18:50	Further hemoglobin decrease	Hgb dropped to 5.7 g/dL.
20:25	Metabolic decompensation	pH 7.12, base deficit −11
20:40	Persistent instability	HR 140 bpm; ongoing hemodynamic instability despite resuscitation
21:45	Post-transfusion response	Hgb improved to 11.0 g/dL after transfusion
23:12	Repeat angiography	No active extravasation identified
ICU course	Refractory shock	Progressive metabolic acidosis, respiratory failure, vasopressor dependence
~26–27 h	Outcome	Cardiac arrest and death

## Discussion

Acute abdominal pain requires rapid exclusion of life-threatening etiologies, including catastrophic vascular emergencies, such as ruptured abdominal aortic aneurysm, aortic dissection with rupture, visceral artery aneurysm rupture, and acute mesenteric ischemia, as well as other emergent intra-abdominal conditions [15]. Other intra-abdominal processes, such as perforated viscus, bowel obstruction with ischemia, and acute pancreatitis, must also be considered [15]. Early risk stratification is essential to guide diagnostic decision-making in the ED [15]. Persistent tachycardia reflects compensatory sympathetic activation and may represent an early marker of significant underlying pathology, including hemorrhage, preceding the development of hypotension [16]. In this context, reliance on initially reassuring laboratory values or the absence of overt shock may be misleading, and advanced imaging should not be delayed [10].

This consideration is particularly important in suspected retroperitoneal pathology. The retroperitoneum is a large anatomical compartment posterior to the peritoneal cavity that contains the kidneys, adrenal glands, pancreas, portions of the duodenum and colon, and major vascular structures including the abdominal aorta and inferior vena cava [11]. Hemorrhage within this space poses unique diagnostic challenges, as it can accommodate large volumes of blood without overt peritoneal signs [10,11]. As a result, patients often present with nonspecific symptoms, such as flank, back, or abdominal pain, and early clinical findings may be subtle or misleading [3,4].

Although ultrasonography is widely used in the emergency setting, its diagnostic utility in this context is limited. It may detect intraperitoneal free fluid but can fail to identify hemorrhage confined to the retroperitoneal space [6,7]. Consequently, contrast-enhanced CT is essential in the evaluation of suspected retroperitoneal bleeding, enabling rapid detection of hemorrhage, accurate delineation of its extent, and identification of the underlying source and vascular involvement [10,11].

While initial resuscitative principles are similar across etiologies of retroperitoneal hemorrhage, important differences exist in definitive management. Large-vessel catastrophes typically require emergent surgical or endovascular repair due to high-volume arterial bleeding and rapid hemodynamic compromise [4]. In contrast, hemorrhage secondary to angiomyolipoma is most often managed with transcatheter arterial embolization, as the bleeding source is typically focal and amenable to targeted endovascular control while preserving uninvolved renal parenchyma [12-14]. This nephron-sparing approach has been associated with technical success rates of approximately 85-97%, avoidance of surgery in up to 96% of cases, and preservation of renal function in the majority of patients [12-14].

Selective and superselective embolization techniques are preferred when feasible, as they allow precise control of bleeding while minimizing loss of functional renal parenchyma [12-14]. In contrast, nonselective embolization may result in greater parenchymal compromise due to proximal vessel occlusion. Additionally, angiomyolipomas may exhibit complex vascular supply, including extra-renal collateralization, which can further complicate both diagnosis and endovascular management [12-14].

In this case, clinical and laboratory deterioration supported ongoing hemorrhage, later confirmed angiographically. Time to definitive hemorrhage control was influenced by the need for transfer to facilitate endovascular intervention. Therefore, early CT evaluation should be strongly considered in patients with abdominal pain and persistent tachycardia, as apparent clinical stability may mask life-threatening retroperitoneal hemorrhage.

## Conclusions

Wünderlich syndrome is a spontaneous, non-traumatic renal hemorrhage confined to the subcapsular or perirenal space, representing a form of retroperitoneal bleeding that may progress rapidly and lead to hemodynamic compromise. Significant retroperitoneal hemorrhage can occur in the early absence of hypotension, with tachycardia serving as an early indicator of underlying pathology. In the emergency setting, abdominal pain accompanied by persistent tachycardia represents a high-risk clinical pattern that should prompt urgent diagnostic evaluation. Reliance on physical examination, initial laboratory values, or point-of-care ultrasonography -even in the absence of hematuria or hypotension- may be insufficient due to the occult nature of retroperitoneal hemorrhage. Early cross-sectional imaging is therefore critical to establish the diagnosis, accurately localize the source of bleeding, and enable timely escalation to definitive management. Maintaining a low threshold for CT in this clinical context is essential to avoid diagnostic delays and improve patient outcomes.
